# WWOX Possesses *N*-Terminal Cell Surface-Exposed Epitopes WWOX_7-21_ and WWOX_7-11_ for Signaling Cancer Growth Suppression and Prevention In Vivo

**DOI:** 10.3390/cancers11111818

**Published:** 2019-11-19

**Authors:** Wan-Jen Wang, Pei-Chuan Ho, Ganesan Nagarajan, Yu-An Chen, Hsiang-Ling Kuo, Dudekula Subhan, Wan-Pei Su, Jean-Yun Chang, Chen-Yu Lu, Katarina T. Chang, Sing-Ru Lin, Ming-Hui Lee, Nan-Shan Chang

**Affiliations:** 1 Laboratory of Molecular Immunology, Institute of Molecular Medicine, College of Medicine, National Cheng Kung University, Tainan 70101, Taiwan; mesha19871001@gmail.com (W.-J.W.); peggy821124@gmail.com (P.-C.H.); nagarajangi@mail.com (G.N.); momofish0716@gmail.com (Y.-A.C.); 100712028@gms.tcu.edu.tw (H.-L.K.); dsubhan71@gmail.com (D.S.); annie1124@gmail.com (W.-P.S.); jeanyunc@gmail.com (J.-Y.C.); chenyulu0226@gmail.com (C.-Y.L.); ktchang@buffalo.edu (K.T.C.); singrulin@gmail.com (S.-R.L.); estilee@gmail.com (M.-H.L.); 2 Advanced Optoelectronic Technology Center, National Cheng Kung University, Tainan 70101, Taiwan; 3 Graduate Institute of Biomedical Sciences, College of Medicine, China Medical University, Taichung 40402, Taiwan

**Keywords:** tumor suppressor, WWOX, epitopes, peptides, phosphorylation, stem cell spheres, Zfra, time-lapse microscopy

## Abstract

Membrane hyaluronidase Hyal-2 supports cancer cell growth. Inhibition of Hyal-2 by specific antibody against Hyal-2 or pY216-Hyal-2 leads to cancer growth suppression and prevention in vivo. By immunoelectron microscopy, tumor suppressor WWOX is shown to be anchored, in part, in the cell membrane by Hyal-2. Alternatively, WWOX undergoes self-polymerization and localizes in the cell membrane. Proapoptotic pY33-WWOX binds Hyal-2, and TGF-β induces internalization of the pY33-WWOX/Hyal-2 complex to the nucleus for causing cell death. In contrast, when pY33 is downregulated and pS14 upregulated in WWOX, pS14-WWOX supports cancer growth in vivo. Here, we investigated whether membrane WWOX receives extracellular signals via surface-exposed epitopes, especially at the S14 area, that signals for cancer growth suppression and prevention. By using a simulated 3-dimentional structure and generated specific antibodies, WWOX epitopes were determined at amino acid #7 to 21 and #286 to 299. Synthetic WWOX7-21 peptide, or truncation to 5-amino acid WWOX7-11, significantly suppressed and prevented the growth and metastasis of melanoma and skin cancer cells in mice. Time-lapse microscopy revealed that WWOX7-21 peptide potently enhanced the explosion and death of 4T1 breast cancer stem cell spheres by ceritinib. This is due to rapid upregulation of proapoptotic pY33-WWOX, downregulation of prosurvival pERK, prompt increases in Ca^2+^ influx, and disruption of the IkBα/WWOX/ERK prosurvival signaling. In contrast, pS14-WWOX7-21 peptide dramatically increased cancer growth in vivo and protected cancer cells from ceritinib-mediated apoptosis in vitro, due to a prolonged ERK phosphorylation. Further, specific antibody against pS14-WWOX significantly enhanced the ceritinib-induced apoptosis. Together, the *N*-terminal epitopes WWOX7-21 and WWOX7-11 are potent in blocking cancer growth in vivo. WWOX7-21 and WWOX7-11 peptides and pS14-WWOX antibody are of therapeutic values in suppressing and preventing cancer growth in vivo.

## 1. Introduction

WW domain-containing oxidoreductase, known as WWOX, FOR and WOX1, was originally designated as a tumor suppressor protein [1,2,3,4,5,6]. Human *WWOX* gene is located on a chomosomal common fragile site 16q23 or *FRA16D*. Null mutations and promoter hypermethylation of *WWOX* gene may result in loss of WWOX protein [1,2,3,4,5,6]. WWOX is crucial in supporting neural development and differentiation. For example, WWOX deficiency in newborns leads to the development of severe neural diseases, growth retardation, metabolic disorders, and early death [7,8,9,10,11]. Accumulating evidence reveals that WWOX limits neurodegeneration [12,13]. Most recently, *WWOX* gene is determined to be a risk factor for Alzheimer′s disease (AD) [14]. In our recent report [15], we determined that p53 blocks WWOX-mediated inhibition of inflammatory immune response (e.g., splenomegaly) caused by cancer in vivo, which leads to protein aggregation in the brain such as in the AD. Although WWOX is considered as a tumor suppressor protein, WWOX-deficient human newborns do not spontaneously develop cancer [5,7,8].

Many review articles have comprehensively addressed the functional properties of WWOX [4,5,6,7,8]. In brief, activated pY33-WWOX binds pS46-p53 for inducing apoptosis from the mitochondria or nuclei in cells [3,4,16,17,18,19]. WWOX prevents p53 from being degraded by ubiquitination and proteasomes in the cytoplasm [16,17,18,19]. Exogenous 17β-estradiol at µM levels induces apoptosis via the WWOX/p53 signaling [20,21]. When WWOX and p53 are dysfunctional, osteosarcoma occurs in a double knockout mouse model [22]. WWOX maintains genomic stability by controlling ATM activation and DNA damage response [23,24]. The interaction of BRCA1 and WWOX supports non-homologous end-joining DNA repair, which is the dominant repair pathway for DNA double-strand breaks in WWOX sufficient cells [25]. WWOX-mediated cancer suppression has recently been established in *Drosophila* [26].

In this study, we explored the functional significance of membrane bound WWOX and its binding partners in cancer suppression. By immunoelectron microscopy [27,28], we determined the presence of a Hyal-2/WWOX complex on the cell surface and its relocation to the nuclei during stimulation of cells with transforming growth factor β TGF-β [27], hyaluronan [28], or under traumatic brain injury in rat [28]. WWOX does not have a membrane localization signal, but it can polymerize by itself on the cell surface [27,28]. The mechanism of the membrane localization is unknown. We hypothesize that WWOX exhibits functional surface-exposed epitopes that sense biological stimuli. For example, WWOX-positive cells migrate collectively, and WWOX-negative cells migrate individually [29]. Upon facing WWOX-positive cells, WWOX-deficient cells promptly run away by retrograde migration, and then induce death of WWOX-positive cells via super induction of redox activity [29]. Later, WWOX-deficient cells compromise with WWOX-positive cells by secreting autologous TGF-β so as to undergo anterograde migration in a collective manner [29]. Both WWOX-positive and -negative cells ultimately merge with each other, even though they are from different species [29]. The observations suggest that membrane WWOX acts as a sensor in coordinating cell-to-cell recognition and communications.

Additionally, pS14-WWOX is frequently accumulated in solid tumors to enhance their growth [30], as well as in AD brain to accelerate disease progression [31]. Suppression of S14 phosphorylation in WWOX by Zfra (zinc finger-like protein that regulates apoptosis) peptide significantly reduces cancer growth in mice [30] and restores memory loss in triple transgenic mice for AD [31]. Zfra peptide binds membrane Hyal-2 [30]. Also, Zfra binds WWOX at the *N*-terminal leader/first WW domain and the *C*-terminal SDR domain [32].

Overall, the aforementioned observations suggest that the *N*-terminal leader sequence before the WW domain is functional, especially at the conserved phosphorylation site S14 and the surrounding area. Presumably, a hydrophilic stretch surrounding S14 is a surface-exposed epitope. Thus, we examined whether WWOX7-21 and other potential areas are cell surface-exposed epitopes, and whether these epitopes participate in cancer growth suppression in vivo. Whether the WWOX7-21 peptide activates the Hyal-2/WWOX/Smad4 signaling for growth suppression is discussed.

## 2. Results

### 2.1. WWOX7-21 and WWOX7-11 Peptides Effectively Inhibit Melanoma Cell Growth in Both Immune Competent and Deficient Mice 

**Figure 1:**
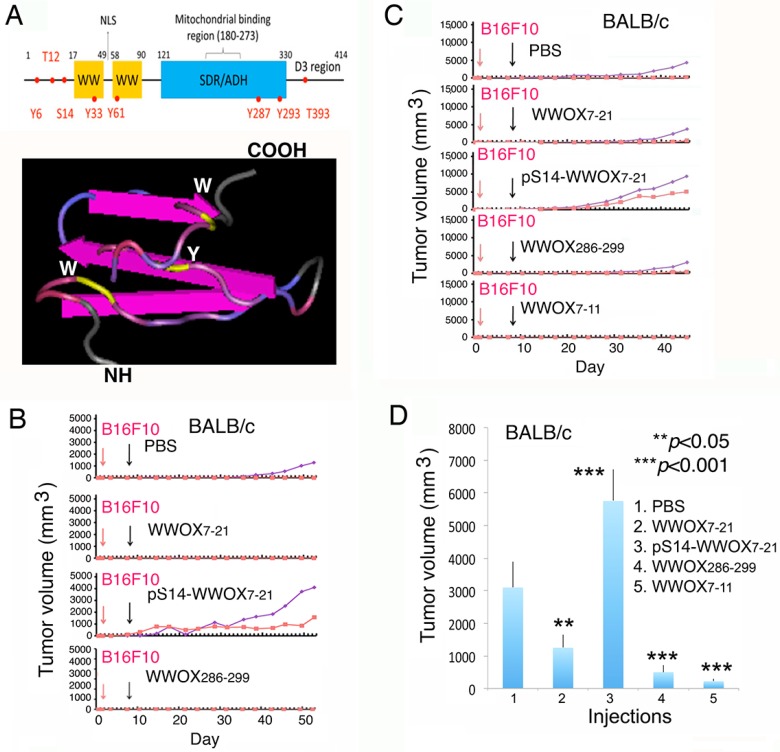
WWOX7-21 and WWOX7-11 peptides suppressed cancer growth in vivo. (**A**) A schematic primary structure of the first WW domain of WWOX is shown. (**B**,**C**) BALB/c mice were inoculated with B16F10 melanoma cells in the left and right flanks (red and blue lines of growth curves). A week after, mice received indicated peptides (1 mM in 100 µL PBS) via tail vein injections. WWOX7-21, WWOX7-11 and WWOX286-299 blocked B16F10 growth. pS14-WWOX7-21 was ineffective. (**D**) The end-point tumor volumes are shown (*n* = 6; mean ± SD).

We analyzed the potential cell surface-exposed epitopes and made WWOX7-21, WWOX7-11, and WWOX286-299 peptides (Figure 1A). The primary structure of WWOX and a simulated tertiary structure of the *N*-terminal head and the adjacent first WW domain are shown (Figure 1A).

We investigated the in vivo effects of WWOX peptides in controlling cancer growth. BALB/c mice were inoculated with melanoma B16F10 cells in both left and right flanks. A week later, mice were treated with sterile phosphate-buffered saline (PBS), WWOX7-21, or pS14-WWOX7-21 peptide (1 mM in 100 µL PBS) via tail vein injections (Figure 1B). Compared to controls, B16F10 growth was significantly enhanced in the pS14-WWOX7-21-treated mice (>2-fold). 

In contrast, WWOX7-21 and WWOX286-299 effectively blocked the melanoma growth (Figure 1B). Indeed, a truncated WWOX7-11 peptide was most effective in blocking melanoma growth in BALB/c mice, as compared to other peptides (Figure 1C). The tumors were allowed to grow greater than 5000 mm^3^ for assessing the inhibitory effect of WWOX7-11 (Figure 1C). Mice were sacrificed on the day 47th, and the end-point data for melanoma growth is shown (Figure 1D).

**Figure 2:**
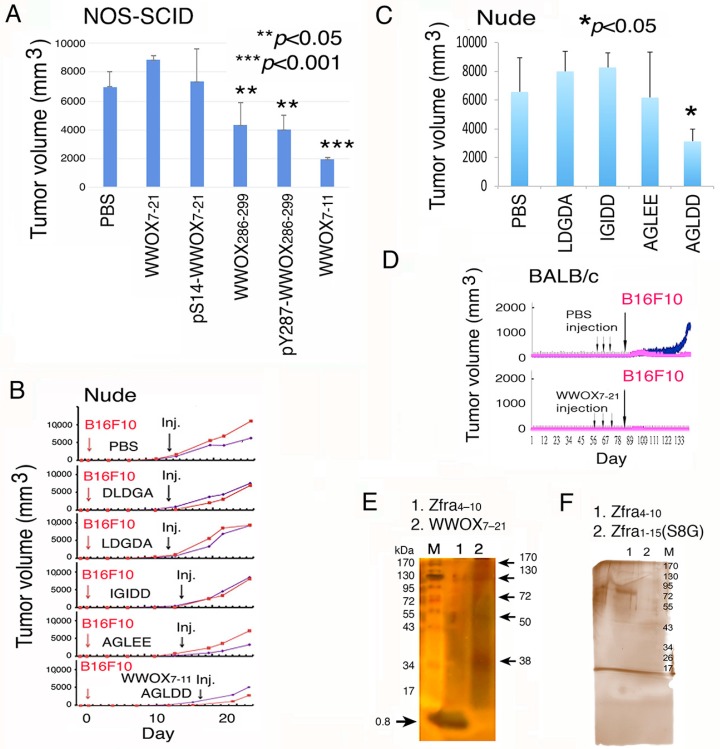
WWOX7-11 peptide is most potent in suppressing cancer growth in vivo (**A**) Each NOD-SCID mouse received an indicated peptide via subcutaneous injection in one side of the flanks, and B16F10 cells inoculated simultaneously in the other side. WWOX7-11 blocked B16F10 growth (*n* = 3; mean ± SD). (**B**,**C**) Scrambled peptides of WWOX7-11 (AGLDD) did not block melanoma growth in nude mice (*n* = 4; mean ± SD). The tumor growth curves and the end-point tumor sizes are shown. (**D**) BALB/c mice received tail vein injections of WWOX7-21 once per week for 3 consecutive weeks, followed by inoculating cancer cells in both flanks. The growth of B16F10 cancer cells was blocked (representative data from 2 experiments). (**E**) Zfra4-10 and WWOX7-11 peptides were resuspended in sterile Milli-Q water (400 µM), and allowed to sit for 3 h in the room temperature. Partial self-polymerization of the peptides is shown. (**F**) Under similar conditions, Zfra4-10 and Zfra1-15(S8G) were resuspended in PBS and incubated at room temperature for 3 h. Zfra4-10 underwent polymerization, and the S8G mutant failed to polymerize.

Instead of tail vein injections, T/B cell-deficient NOD-SCID mice received an indicated peptide in one side of the flanks via subcutaneous injections, and B16F10 cells inoculated simultaneously in the other side. As summarized from end-point experiments, WWOX7-11, WWOX286-299 and pY287-WWOX286-299 peptides significantly blocked melanoma growth (Figure 2A).

In control experiments, scrambled peptides for WWOX7-11 (AGLDD) were designed and synthesized, including DLDGA, LDGDA, IGIDD, and AGLEE. All scrambled peptides were infective in blocking B16F10 melanoma growth in T cell-deficient nude mice (Figure 2B,C). The graphs show the tumor growth curves and the end-point of tumor sizes (Figure 2B,C). In appropriate controls, mice received injections with PBS. In these experiments, tumors grew up to ~200 mm^3^ in mice, prior to tail veins injections with scrambled peptides. Also, tumors were allowed to grow up to ~500 mm^3^ in mice, followed by treating with WWOX7-11 peptide. The data showed the effectiveness of the WWOX7-11 peptide in tumor growth inhibition (Figure 2B,C).

### 2.2. WWOX7-21 Peptide Effectively Prevents Skin Cancer and Melanoma Cell Growth In Vivo

For cancer prevention, BALB/c mice, respectively, received 3 injections of WWOX7-21 peptide via tail veins over three consecutive weeks. These mice effectively resisted the growth of inoculated skin B16F10 cells by greater than 85% (Figure 2D). Optimal self-polymerization is essential for Zfra-mediated anticancer activity [30]. Ser8 and Cys9 contribute to the Zfra self-polymerization. Alteration of Ser8 to Gly8 abolishes the self-polymerization of Zfra and its anticancer function [30]. When both Zfra4-10 and WWOX7-21 peptides were suspended in sterile Milli-Q water, partial polymerization of both peptides occurred (Figure 2E). Like Zfra [30], polymerization of WWOX7-21 peptide positively correlates with its anticancer function. In agreement with our previous report [30], Zfra4-10 and Zfra1-15 underwent polymerization effectively in PBS (Figure 2F; data not shown for Zfra1-15). Alteration of S8 to G8 resulted in failure of polymerization (Figure 2F).

### 2.3. WWOX7-21 Peptide Blocks Melanoma Cell Metastasis

**Figure 3:**
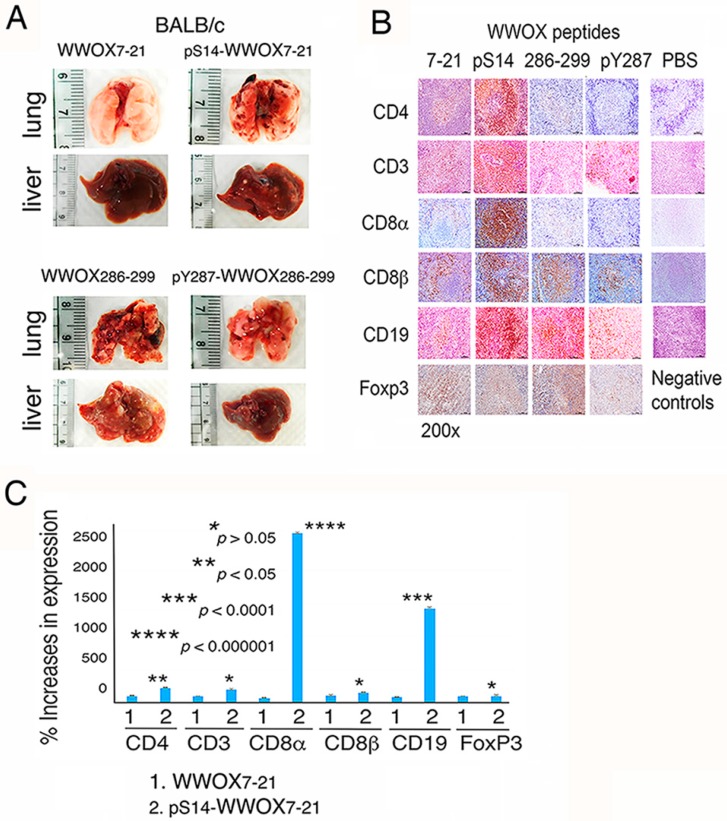
WWOX7-21 peptide suppresses melanoma metastasis, whereas pS14-WWOX7-21 peptide induces cytotoxic T cell expansion and fails to block cancer metastasis in vivo. (**A**) BALB/c mice received subcutaneous injections of WWOX7-21 peptide and became resistant to the metastasis of melanoma B16F10 cells to the lung and liver. Other peptides were ineffective. (**B,C**) pS14-WWOX7-21 peptide significantly induced the expansion of CD4^+^ and CD8α^+^ T and CD19^+^ B cells in the germinal centers, but had no effect on Foxp3^+^ Treg cells in BALB/c mice. Scale bar = 50 µm.

We investigated whether WWOX peptides affect cancer cell metastasis and immune cell expansion. BALB/c mice were injected subcutaneously with an indicated WWOX peptide in one side of the flank, and simultaneously with melanoma B16F10 cells in the other side. Control mice receiving PBS died within two months due to enormous tumor growth and metastasis, and the internal organs decomposed. Compared to pS14-WWOX7-21, WWOX286-299, and pY287-WWOX286-299 peptides, WWOX7-21 was most effective in blocking B16F10 metastasis to the lung and liver (Figure 3A). Similarly, when breast cancer 4T1 cells were inoculated in BALB/c mice, WWOX7-21 peptide was most effective in blocking cancer growth and metastasis (data not shown).

### 2.4. pS14-WWOX7-21 Peptide Dramatically Induces Cytotoxic T Cell Expansion but Fails to Block Cancer Cell Metastasis

pS14-WWOX7-21 peptide failed to block melanoma cell metastasis and yet induced significant upregulation of the expansion of spleen helper CD4^+^ T cells (2-fold increase), cytotoxic CD8α^+^ T cells (25-fold increase) and CD19^+^ B cells (14-fold increase) in the germinal centers (Figure 3B,C). WWOX7-21, WWOX286-299, and pY287-WWOX286-299 peptides had no effect on T/B cell expansion. Foxp3^+^ T regulatory (Treg) cells were not responsive to the induction by all the peptides (Figure 3B,C). Together, dramatic upregulation of cytotoxic CD8α^+^ T cells, along with CD19^+^ B cells, by the pS14-WWOX7-21 peptide did not lead to melanoma growth suppression.

### 2.5. WWOX7-21 and WWOX286-299 Peptides Bind Cell Surface and Colocalize with Membrane Type II TGF-β Receptor (TβRII)

**Figure 4:**
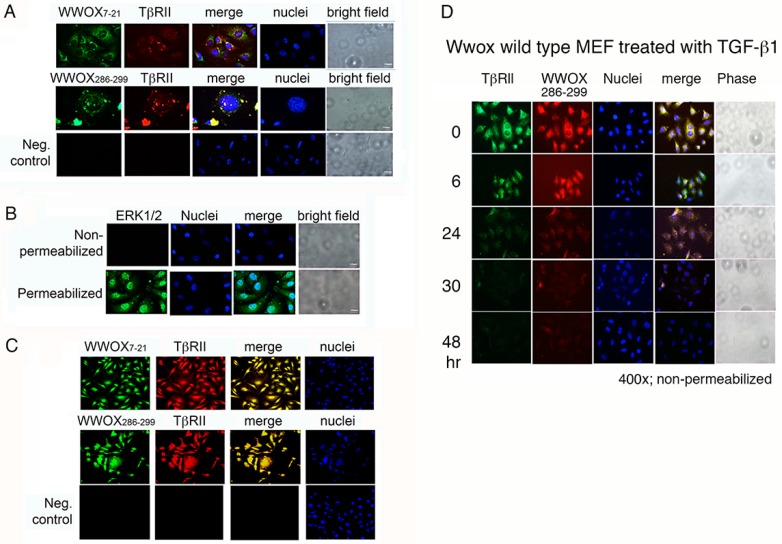
WWOX7-21 and WWOX286-299 peptides colocalize with cell membrane type II TGFβ receptor (TβRII). (**A**) WWOX-negative MDA-MB-231 cells were incubated with WWOX7-21 or WWOX286-299 peptide at 4 °C for 30 min, followed by processing immunostaining using our generated peptide antibodies. These peptides were shown to colocalize with membrane TβRII. Cells were not permeabilized with Triton X-100. In the negative controls, no primary antibodies were used. (**B**) In control experiments, cells were stained for ERK1/2 to show its nuclear localization in Triton X-100-permeabilized cells. No signal was shown in non-permeabilized cells. (**C**) Colocalization of TβRII with WWOX7-21 and WWOX286-299 is shown in MDA-MB-231 cells, as determined by confocal microscopy. (Magnification 400×). (**D**) *Wwox*^+/+^ wild type MEF cells were pretreated with WWOX286-299 peptide for 5 min at 37 °C, followed by treating with TGF-β1 (10 ng/mL) for indicated times. The WWOX286-299 peptide colocalized with TβRII, and TGF-β1 appeared to induce internalization of the peptide/TβRII complex.

We examined whether WWOX peptides adhere to a specific protein(s) on cancer cell surface and thereby signal for cancer cell survival or death. We verified the presence of the exogenous peptides on cell membrane by immunofluorescence staining using specific homemade antibodies [12,16,17,33]. Live cells were used for immunostaining without permeabilization. WWOX-negative breast MDA-MB-231 cells were incubated with WWOX7-21 or WWOX286-299 peptide at 4 °C for 30 min, and then subjected to immunostaining using homemade peptide antibodies [12,16,17,33]. Colocalization analysis showed that the peptides are located in the cell surface and colocalize with membrane TβRII (Figure 4A). In negative controls, primary antibodies were not added and the immunostaining showed negative results (Figure 4A). In control experiments, permeabilized cells were positive for nuclear localization of ERK1/2 (Figure 4B). No signal was observed in non-permeabilized cells (Figure 4B). Finally, confocal microscopy showed the colocalization of TβRII with WWOX7-21 and WWOX286-299 peptides in MDA-MB-231 cells (Figure 4C).

The peptides may be internalized together with TβRII. For example, *Wwox*^+/+^ wild type MEF cells were pretreated with WWOX286-299 peptide for 10 min at 4°C, followed by culturing at 37 °C for 5 min and treating with TGF-β1. Colocalization of WWOX286-299 peptide with TβRII was shown (Figure 4D). Disappearance of the WWOX286-299 peptide and TβRII from the cell surface occurred with time, suggesting that the peptide binds TβRII, and TGF-β1 induces internalization of the peptide/TβRII complex.

### 2.6. WWOX Is Clustered in the Cell Membrane

From the observations shown above (Figure 4), WWOX7-21 and WWOX286-299 peptides are the potential cell surface-exposed epitopes in WWOX. By using the generated antibodies, live non-permeabilized colon cancer HCT116 cells were stained with each indicated antibody at 4 °C for 30 min, and then stained with membrane-specific red-fluorescent LavaCell. WWOX is shown to cluster on the cell surface (Figure S1). WWOX is known to bind and cluster together with membrane hyaluronidase Hyal-2 [5,27,28]. These antibodies were against WWOX, respectively, at the *N*-terminal amino acid #20 to 42, with or without Y33 phosphorylation, and at the *C* terminal amino acid #286 to 299, with or without Y287 phosphorylation. Similar results were observed by testing squamous cell carcinoma SCC4 cells (Figure S2). pY33 is in the first WW domain, pY61 in the second WW domain, and pY287 in the SDR domain. pT393 is located in the *C*-terminal D3 tail [4,5,8,33,34,35].

### 2.7. Ceritinib Mediates 4T1 Cell Sphere Shinkage (Pre-Explosion Stage) and then Explosion and Death (Explosion Stage)

Expanding solid tumors exhibit significant accumulation of prosurvival pS14-WWOX protein, but the levels of anticancer pY33-WWOX are reduced [30]. Switching from Y33 to S14 phosphorylation in WWOX enhances disease progression in vivo, including cancer and Alzheimer′s disease [30,31]. 

The cancer stem cells in the spheres are highly resistant to death by chemotherapeutic drugs [35,36]. To further validate the role of pS14-WWOX in supporting cancer survival, we measured the extent of cell death in the 4T1 breast cancer stem cell spheres for their uptake of PI, as determined by time-lapse microscopy [28,33,37,38]. Cancer stem cell spheres frequently express pS14-WWOX and stem cell markers in their inner mass, but not in the outer surface (Figure S3).

**Figure 5:**
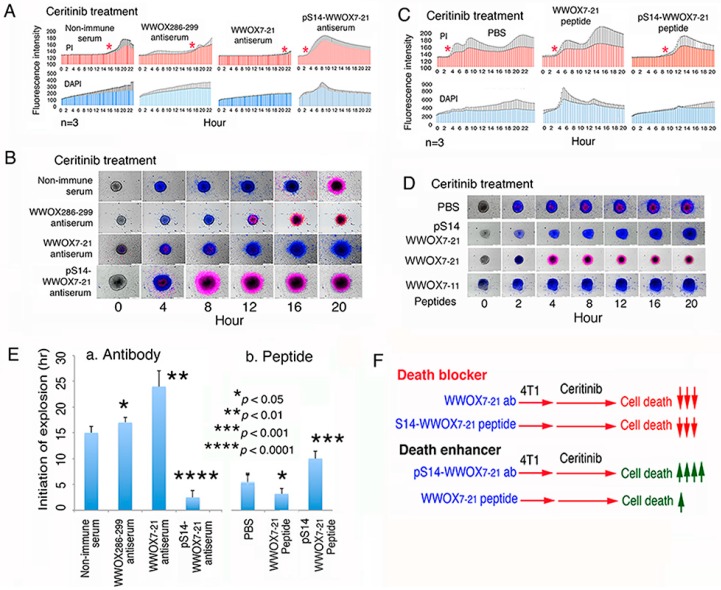
pS14-WWOX7-21 peptide blocked ceritinib-mediated 4T1 stem cell sphere explosion and death. (**A**,**B**) 4T1 stem cell spheres were treated with an indicated antiserum (1/100 dilution) for 30 min, and then exposed to ceritinib (30 µM) for time-lapse fluorescent microscopy at 37 °C. DAPI uptake in the nuclei (blue) by live cells indicates an increased nuclear membrane permeability, and PI uptake by nuclei (red) indicates cell death (mean ± SD; *n* = 3; SD in black shaded areas). The red stars show the initiation of stem cell sphere explosion and death. Representative changes in sphere morphology with time are shown. (**C**,**D**) pS14-WWOX7-21 peptide strongly blocked ceritinib-mediated cell death and sphere explosion; however, WWOX7-21 peptide drastically enhanced the cell death and sphere explosion event. (**E**) The time of initiation of 4T1 cell sphere explosion is shown (*n* = 3). (**F**) Summary of enhancers and inhibitors for ceritinib-mediated cell death.

When 4T1 cells, including cell spheres and surrounding individual cells, were pretreated with 10 µL of non-immune serum (1:100 dilution) for 30 min, followed by treating with a chemotherapeutic drug ceritinib (30 µM), cells started to die within 12 h, as determined by PI uptake (Figure 5A,B; Supplementary Video S1). Ceritinib is an inhibitor of anaplastic lymphoma kinase (ALK) [39]. The spheres initially shank, then rapidly picked up DAPI, and simultaneously sucked in the surrounding cells (12–15 h), followed by explosion (>81 h) and death with propidium iodide (PI) uptake. In general, there are two main stages in the ceritinib-mediated 4T1 breast cancer stem cell sphere explosion and death. In response to ceritinib, 4T1 cell spheres underwent shinkage (pre-explosion stage) and then explosion and death (explosion stage) (Figure S4; Video S1).

### 2.8. Treatment of 4T1 Cells with pS14-WWOX Antibody Accelerates Ceritinib-Mediated Sphere Explosion and Cell Death 

Antiserum against WWOX286-299 (located at the *C*-terminal SDR domain) did not block the ceritinib-mediated cell death (Figure 5A,B; Video S2). Pre-exposure of 4T1 cells to antiserum against the *N*-terminal epitope of WWOX7-21, followed by exposure to ceritinib, resulted in dramatic inhibition of cell death (>90%; Figure 5A,B; Video S3). Cells in the spheroids rapidly picked up the nuclear stain DAPI in less than 30 min but did not die (Figure 5A,B; Video S3), suggesting that ceritinib increases the nuclear membrane permeability. In stark contrast, pre-treatment of 4T1 cells with an aliquot of pS14-WWOX7-21 peptide antiserum, followed by exposure to ceritinib, resulted in rapid acceleration of sphere explosion/cell death in less than 4 h (>90%; Figure 5A,B), suggesting that endogenous pS14-WWOX is protective against cell death. The peptide antibody recognizes the full-length endogenous pS14-WWOX protein [33].

### 2.9. pS14-WWOX Peptide Protects 4T1 Cells from Ceritinib-Mediated Death In Vitro

In contrast, when 4T1 cells were pretreated with synthetic pS14-WWOX7-21 peptide for 30 min at 37°C, ceritinib-mediated sphere explosion/cell death was dramatically delayed (>20 h; Figure 5C,D). No sphere explosion was observed for more than 48 h (Figure 5D). In controls, ceritinib alone induced sphere explosion/cell death in 5 to 6 h or less (Figure 5C,D). In contrast to the observation using WWOX7-21 antibody (Figure 5A,B), WWOX7-21 peptide accelerated sphere explosion/cell death in approximately 3 h (Figure 5C,D), suggesting that exogenous WWOX7-21 peptide, together with membrane WWOX, Hyal-2 or TβRII, is sufficient in inducing apoptosis.

We summarized the time needed for the initiation of 4T1 stem cell sphere explosion using the aforementioned peptides and antibodies (Figure 5E). Both WWOX7-21 peptide and pS14-WWOX7-21 antibody enhanced cancer cell death, whereas WWOX7-21 antibody and pS14-WWOX7-21 peptide supported cancer cell growth (Figure 5F).

In parallel experiments, we carried out the 4T1 sphere explosion/death assay at room temperature. We reported that when normal or cancer cells are subjected to UV irradiation and subsequent cold shock, the cells undergo bubbling cell death (BCD), in which the cell death initiates from the nucleus [5,19,37,38]. Bubbling cell death does not belong to the death event as shown in apoptosis and necroptosis. 4T1 spheres were exposed to ceritinib for imaging by time-lapse microscopy at room temperature (Figure S5A; Video S4). Again, under similar conditions, pS14-WWOX7-21 peptide strongly blocked ceritinib-mediated 4T1 sphere explosion and death (Figure S5A), and WWOX7-21 accelerated the cell death (Figure S5A; Video S5). WWOX7-21 peptide was truncated to WWOX7-11, and the peptide did not enhance ceritinib-mediated 4T1 sphere explosion and death (Figure S5A). When the temperature was set at 37 °C, pS14-WWOX7-21 peptide strongly blocked ceritinib-mediated sphere explosion and death (Figure S5B).

### 2.10. Ceritinib Upregulates Proapoptotic pY33-WWOX and Meanwhile Induces Ca^2+^ Influx for Leading to Apoptosis of 4T1 Cells

**Figure 6:**
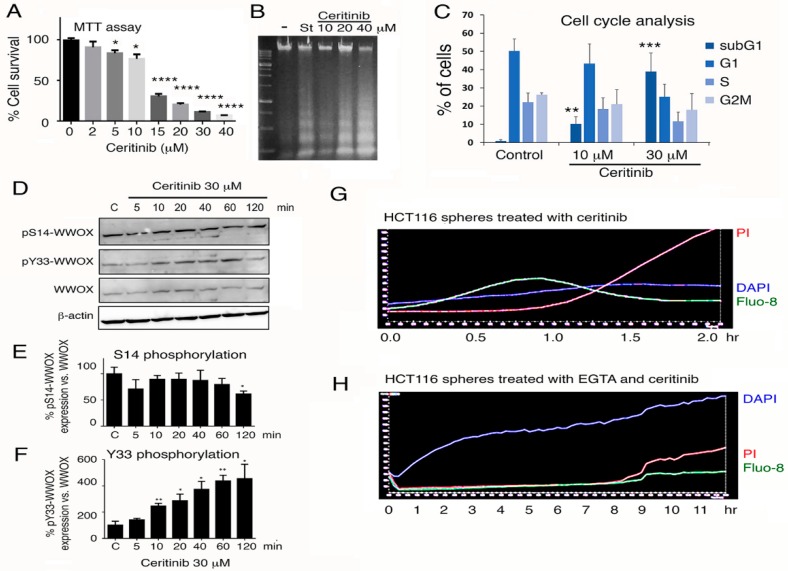
Ceritinib induces 4T1 cell apoptosis by inducing calcium influx and upregulating pY33-WWOX. (**A**–**C**) 4T1 cells were treated with an indicated concentration of ceritinib for 24 h at 37 ℃ and were subjected to MTT assay for cell viability (*n* = 3, * *p* < 0.05; **** *p* < 0.0001) (**A**), and apoptosis by DNA fragmentation analysis, where staurosporine (St) treatment is regarded as a positive control (**B**), and cell cycle analysis (**C**). Note that ceritinib induced apoptosis by increasing the percentages of SubG1 phase (*n* = 3, ** *p* < 0.01; *** *p* < 0.001). (**D**–**F**) When 4T1 cells were treated with ceritinib for indicated times at 37 °C, decreased S14 phosphorylation but increased Y33 phosphorylation in WWOX was observed. Quantification of protein expression was normalized to β-actin, and then normalized to the control. (*n* = 3, * *p* < 0.05; ** *p* < 0.01). (**G**,**H**) Ceritinib rapidly induced Ca^2+^ influx (green fluorescent Fluo-8) and simultaneous DAPI uptake (blue fluorescence) in 4T1 cells in 20 min prior to cell death (PI uptake; red fluorescence). Exposure of 4T1 cells to EGTA for 10 min prior to treating with ceritinib resulted in retarded cell death and abolished Ca^2+^ influx, whereas DAPI uptake was enhanced.

We examined how ceritinib induces 4T1 cell death. Ceritinib significantly reduced cell viability in a dose-related manner, as determined by MTT assay (Figure 6A). Under similar conditions, ceritinib induced DNA fragmentation in a dose-related manner (Figure 6B). Cell cycle analysis also revealed that ceritinib increased the subG1 phase of the cell cycle (Figure 6C), indicating that ceritinib induces apoptosis of 4T1 cells. Next, we examined whether ceritinib activates WWOX via Y33 phosphorylation [16,17]. When 4T1 cells were treated with ceritinib, endogenous WWOX rapidly underwent phosphorylation at Y33, but not at S14, in 10 min (Figure 6D–F). Additionally, we showed that ceritinib rapidly caused Ca^2+^ influx and simultaneously stimulated DAPI uptake in 4T1 cells in 20 min, followed by cell death. When 4T1 cells were treated with EGTA for 10 min and then exposed to ceritinib, these treated cells exhibited retarded Ca^2+^ influx and cell death (Figure 6G,H).

### 2.11. WWOX Peptides Counteract the Ceritinib-Mediated Apoptosis via Regulating ERK Phosphorylation

**Figure 7:**
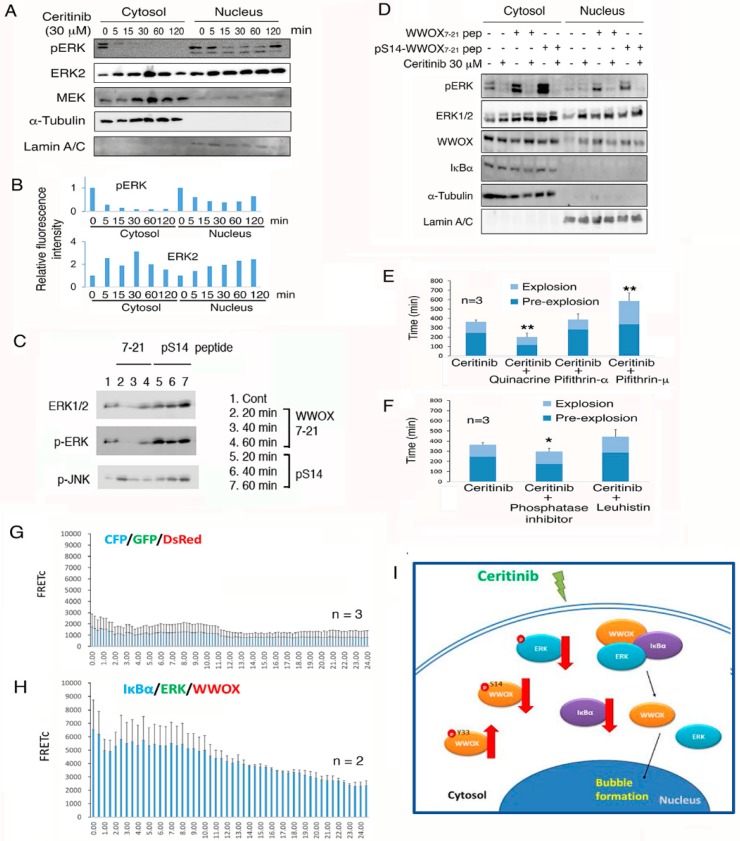
WWOX peptides counteract the ceritinib-mediated apoptosis via regulating ERK phosphorylation and IkBα/WWOX/ERK signaling. (**A**,**B**) Ceritinib rapidly suppressed the phosphorylation of ERK1/2 in 4T1 cells. A time-related nuclear accumulation of ERK2 is shown. Similar results were observed for ERK1/2 using specific antibodies. (**C**) Compared to WWOX7-21 peptide, pS14-WWOX7-21 peptide sustained phosphorylation of ERK and JNK in 4T1 cells. (**D**) 4T1 cells were treated with an indicated peptide (30 μM) for 30 min, and then exposed to ceritinib (30 μM) for 60 min, prior to processing cytosolic and nuclear fractionation and Western blotting. (**E**) 4T1 cells were cotreated with ceritinib (30 μM) and an indicated chemical for time-lapse microscopy. p53 inhibitor pifithin-μ and -α (50 μM) retarded the ceritinib-mediated sphere explosion and apoptosis. p53 activator quinacrine (50 µM) accelerated ceritinib-mediated apoptosis. (**F**) Phosphatase inhibitors (10 μL) enhanced ceritinib-mediated sphere explosion and apoptosis, whereas protease inhibitor leuhistin (30 μM) marginally retarded the effect of ceritinib (*n* = 3, ** *p* < 0.05; * *p* < 0.1). (**G**,**H**) COS7 cells were transiently transfected with ECFP-IkBα, EGFP-ERK and DsRed-WWOX cDNA expression constructs. By thee protein/protein time-lapse FRET microscopy, ceritinib induced the signaling from IkBα to ERK and then to WWOX via energy transfer. In controls, no signaling is observed for ECFP, EGFP and DsRed. (**I**) A schematic graph for ceritinib signaling is shown, namely upregulation of pY33-WWOX, downregulation of p-ERK, dissociation of the IkBα/WWOX/ERK complex, and nuclear translocation of pY33-WWOX to cause apoptosis. Additionally, ceritinib induces cancer stem cell sphere explosion and death. Ceritinib-mediated apoptosis of single cells is switched to bubbling cell death at room temperature.

When 4T1 cells were treated ceritinib, phosphorylation of ERK was rapidly suppressed in 5 min in both cytoplasm and nucleus (Figure 7A,B). A time-related nuclear accumulation of ERK2 is shown (Figure 7A,B). Compared to the WWOX7-21 peptide, pS14-WWOX7-21 peptide rapidly increased and sustained the phosphorylation of ERK and JNK (Figure 7C). Next, 4T1 cells were treated with the WWOX7-21 peptide or pS14-WWOX7-21 peptide for 30 min to induce ERK phosphorylation, followed by treating with ceritinib for 60 min. Western blotting analysis showed that ceritinib effectively suppressed ERK phosphorylation by greater than 90% (Figure 7D).

### 2.12. Endogenous p53 and Aminopeptidase M Enhance Ceritinib-Mediated Cell Sphere Explosion and Cell Death

We continued to investigate the molecular mechanisms by which ceritinib induces sphere explosion and death. When 4T1 cell spheres were exposed to a p53 inhibitor pifithin-μ or pifithin-α and then treated with ceritinib, the apoptosis function of ceritinib was significantly retarded (Figure 7E). In contrast, when endogenous p53 was activated by quinacrine, ceritinib effectively mediated 4T1 cell sphere explosion and death (Figure 7E). A cocktail of phosphatase inhibitors enhanced the ceritinib-induced sphere explosion and death (Figure 7F). Leuhistin, an inhibitor for aminopeptidase M, retarded the effect of ceritinib (Figure 7F). Together, these observations suggest that p53 and aminopeptidase M support the ceritinib-mediated sphere explosion and death, whereas phosphatases counteract the effect.

### 2.13. Ceritinib Suppresses the Prosurvival IkBα/ERK/WWOX Signaling to Cause Cell Death

Certinib suppresses ERK phosphorylation, suggesting that ERK-mediated cell survival is blocked. We investigated whether ceritinib alters the prosurvival IkBα/ERK/WWOX signaling to cause cell death [33,34,37]. 4T1 cells were transfected with ECFP-IkBα, EGFP-ERK, and DsRed-WWOX by electroporation and cultured for 24 to 48 h to determine the tri-molecular binding interactions [34,37]. Ceritinib did not induce the signaling in control cells expressing ECFP, EGFP and DsRed (Figure 7G). In contrast, ceritinib rapidly induced the IkBα/ERK/WWOX signaling in less than 2 h, followed by reduction or complex dissociation (Figure 7H), In summary, ceritinib mediated cell death by downregulating the cytoprotective pS14-WWOX and the prosurvival IkBα/ERK/WWOX signaling, but upregulating the proapoptotic pY33-WWOX protein expression (Figure 7I). Furthermore, ceritinib induces dissociation of the IkBα/ERK/WWOX complex and leads to nuclear translocation of pY33-WWOX and ERK.

## 3. Discussion

### 3.1. pY33 Switching to pS14 for Cancer Promotion in WWOX

We have recently demonstrated that when WWOX phosphorylation at Y33 is switched to S14, pS14-WWOX supports T cell differentiation [33,34], dramatically enhances cancer growth [30], and promotes the progression of neurodegeneration [31]. To further validate this scenario, here we utilized synthetic peptides and demonstrated a crucial cell surface epitope WWOX7-21, which mimics the functions of endogenous WWOX protein in the anticancer response. The WWOX7-21 peptide suppresses and prevents the growth of breast cancer and melanoma cells in vivo, and sensitizes cancer cell sensitivity to ceritinib-mediated apoptosis. WWOX7-21 peptide is further reduced to 5-amino-acid WWOX7-11, and this peptide is even more effective in suppressing cancer growth in vivo. The mechanisms by which both WWOX7-21 and WWOX7-11 peptides suppress cancer growth are unknown and remains to be established.

### 3.2. Role of TβRII in Anchoring WWOX Peptides

Whether membrane-localized WWOX binds TβRII is unknown. Our WWOX peptides, including WWOX7-21, pS14-WWOX7-21, WWOX286-299, and pY287-WWOX286-299, co-localize with TβRII. These peptides may bind membrane WWOX, Hyal-2 and TβRII, and TGF-β1 causes relocation of the complex to the nucleus to exert growth suppression or promotion. For example, WWOX7-21 peptide may physically bind the membrane Hyal-2/WWOX complex to undergo nuclear relocation, together with Smad4, for blocking cancer cell growth [5,27,28].

### 3.3. WWOX7-21 and pS14-WWOX7-21 Peptides Recapitulate the Functional Properties of Endogenous WWOX

While small peptides do not possess tertiary structures, we have previously reported that Zfra4-10 undergoes self-polymerization and binds the cell surface Hyal-2 [30]. This binding results in signaling together with WWOX and Smad4 to block cancer growth [30] and inhibit the progression of neurodegeneration [31]. Here, we showed that both Zfra4-10 and WWOX7-21 peptides undergo aggregation. That is, the WWOX7-21 epitope may contribute, in part, to the capping or clustering of endogenous WWOX in the cell surface. WWOX participates in the control of cell migration [15,29]. The clustered WWOX may act as a molecular sensor in the cell surface to coordinate the recognition and direct the moving of upcoming cells, either from the same or different species of cells ([29] and unpublished).

In stark contrast, when S14 is phosphorylated, endogenous WWOX loses its proapoptotic function in blocking cancer growth [30] and slowly enhancing neurodegeneration [31]. In parallel experiments, pS14-WWOX7-21 peptide was made and shown to strongly increase cancer growth in vivo and blocks ceritinib-mediated cancer cell apoptosis in vitro. pS14-WWOX7-21 peptide strongly induces the expression of cytotoxic CD8α^+^ T cells, but these cells fail to suppress cancer growth. This peptide mimics the functional property of endogenous pS14-WWOX [30,31]. Overall, both WWOX7-21 and pS14-WWOX7-21 peptides recapitulate the functional properties of endogenous WWOX, with or without S14 phosphorylation.

### 3.4. Zfra Induces the Hyal-2/WWOX/Smad4 Signaling for Cancer Suppression

In a non-canonical pathway, TGF-β and hyaluronan bind membrane hyaluronidase Hyal-2 to signal together with WWOX and Smad4 for controlling cell proliferation, migration and death [27,28,30,31,40]. The induced cell death is from over induction of SMAD promoter activation in the nucleus [27]. Accordingly, when cells are overexpressed with the signaling complex protein Hyal-2, WWOX and Smad4, hyaluronan effectively induce a non-apoptotic bubbling cell death [28]. We also reported that treatment of cancer cells with Hyal-2 or pY216-Hyal-2 antibody results in suppression of cancer growth in vivo [41]. In addition, exogenous Zfra peptide binds membrane Hyal-2, and this binding appears to activate the Hyal-2/WWOX/Smad4 signaling in a novel CD3-CD19-Hyal-2^+^ Z lymphocyte for activation and suppression of cancer growth in vivo [30,42,43] and restoration of memory loss in the brain of triple transgenic mouse for Alzheimer′s disease [31].

### 3.5. WWOX Peptides and Their Anticancer Activities in Immune Competent and Deficient Mice

In principle, we have developed anticancer peptides WWOX7-21 and WWOX7-11 and pS14-WWOX antibody. These reagents are of potential use in cancer treatment. Zfra binds membrane Hyal-2 [5,30], and also binds the *N*-terminal WW domain and the *C*-terminal SDR domain of WWOX [32], suggesting that Zfra initiates the Hyal-2/WWOX/Smad4 signaling directly from WWOX and Hyal-2. Zfra is known to prevent and suppress the growth of many types of cancer cells [30,31]. As mentioned above, WWOX7-21 and WWOX7-11 peptides probably drive the Hyal-2/WWOX/Smad4 signaling for cancer suppression by binding with membrane WWOX and/or Hyal-2. Thus, we are exploring whether the peptide-induced ERK activation is related to the Hyal-2/WWOX/Smad4 signaling [27,28], or the prolonged ERK activation is associated with the prosurvival IkBα/WWOX/ERK signaling [33,34].

When mice receive Zfra peptide injection from the tail veins, spleen Z cell activation occurs most effectively in the immune competent BALB/c mice and T cell-deficient nude mice, but not in the T/B-deficient NOD/SCID mice [30]. It is possible that Z cell activation requires the activation of B cell. However, when spleen cells are isolated from NOD/SCID mice and then exposed to Zfra overnight in culture followed by Z cell purification by cell sorting, the purified Z cell is potent in killing cancer cells. Thus, the reduced activity of Zfra in causing Z cell activation in NOD/SCID mice is probably due to failure of access of Zfra to Z cell in the spleen. Whether WWOX_7-21_ and WWOX_7-11_ peptides exhibit similar mechanisms in activating Z cell for attacking cancer cells is unknown and is being tested.

### 3.6. pS14-WWOX7-21 Peptide Induces the Expansion of Spleen CD8α^+^ T and CD19^+^ B Cells

Whether the synthetic WWOX7-21 and WWOX7-11 peptides activate novel immune cells to suppress melanoma and breast cancer cell growth and metastasis in vivo, and sensitize cancer cells to ceritinib cytotoxicity in vitro are unknown. In stark contrast, pS14-WWOX7-21 peptide induces the expansion of spleen CD8α^+^ T and CD19^+^ B cells and dramatically enhances cancer growth in vivo. That is, pS14-WWOX7-21 peptide protects breast cancer 4T1 cells from anticancer drug ceritinib-mediated death. Suppression of pS14-WWOX by specific antibody renders cancer cell sensitive to ceritinib-mediated cytotoxicity. Overall, the observations suggest a conundrum regarding the functional failure of cytotoxic CD8α^+^ T in killing cancer cells during chemotherapy.

### 3.7. pS14-WWOX7-21 Peptide Probably Drives the IkBα/WWOX/ERK Signaling for T/B Cell Maturation

We reported the stepwise maturation of T cell via utilization of the IkBα/WWOX/ERK pathway [33]. During the immune cell maturation process, WWOX undergoes Tyr33 de-phosphorylation first and then Ser14 phosphorylation. pS14-WWOX co-translocates with p-ERK to the nucleus to induce cell maturation [33,34,35]. That is, pS14-WWOX7-21 peptide is likely to drive the IkBα/WWOX/ERK signaling for T/B cell maturation. Failure of CD8α^+^ T cells in blocking cancer growth is probably due to the cell not functionally activated.

### 3.8. The Complex of pY33-WWOX and Hyal-2 Causes Apoptosis in the Nucleus

The relationship between membrane Hyal-2 and WWOX is intriguing. Substantial evidence has demonstrated that pY33-WWOX is potent in causing apoptosis of cancer cells and damaged normal cells such as injured neurons [3,4,5,15,16,17,18]. Alteration of Y33 to R33 or F33 results in the loss of the apoptosis-inducing function of WWOX [3,16,17]. Functional pY33-WWOX binds membrane Hyal-2 [27,28]. Whether pY216-Hyal-2 is necessary for binding with pY33-WWOX is unknown. Presumably, pY216-Hyal-2 temporarily inactivates and protects pY33-WWOX from being degraded by the ubiquitin/proteasome system. When cells were stimulated with TGF-β, the pY33-WWOX/Hyal-2/Smad4 complex translocates to the nucleus and induces apoptosis due to super induction SMAD promoter activation [5,27,28]. During traumatic brain injury, accumulation of the pY33-WWOX/Hyal-2 complex in the apoptotic nucleus correlates with neuronal death in rat brain [28]. High molecular weight hyaluronan is not pro-apoptotic. However, hyaluronan induces apoptosis in cells overexpressing WWOX, Hyal-2 and Smad4 [28].

### 3.9. Ceritinib Upregulates pY33-WWOX, Downregulates p-ERK, Induces Ca^+2^ Influx, and Ultimately Generates DNA Fragmentation 

How ceritinib induces cell death was investigated. Ceritinib is potent inhibitor for anaplastic lymphoma kinase, and this drug is intended primarily for the treatment of metastatic non-small cell lung cancer (NSCLC) [39]. Ceritinib rapidly upregulates proapoptotic pY33-WWOX, downregulates prosurvival p-ERK, promptly increases Ca^+2^ influx, and induces chomosomal DNA fragmentation. The whole event for individual cells is a typical apoptosis. In addition, p53 and aminopeptidase M support the ceritinib-mediated sphere explosion and death, whereas phosphatases counteract the effect. In other words, the ceritinib-induced apoptosis event requires participation of p53, proteases and phosphorylation in many proteins. In the 4T1 stem cell spheres, ceritinib rapidly increases the permeability of nuclear membrane in 4T1 cells, followed by death via Ca^+2^ influx, bubble formation, and subsequent soma explosion.

## 4. Materials and Methods

### 4.1. Cell Lines

Cell lines from ATCC (Manassas, VA, USA) have been maintained in our laboratory and used in the experiments, including (1) human breast cancer MDA-MB-231 [19], (2) mouse melanoma B16F10 [30,31], and (3) mouse breast 4T1-Luc [30,31].

### 4.2. Structure Simulation and Peptide Synthesis

By searching “Structure” for WW domain under PubMed, many established 3D structures are shown. We downloaded the Cn3D software program to view and analyzed the selected sequences. Those sequences, which have the highest identities to that of the first WW domain of human WWOX, were used for amino acid replacement. That is, the original amino acid sequence of WW domain was replaced with the sequence of the first WW domain. A new putative structure was then generated.

To identify potential epitopes, WWOX peptides were synthesized based upon: (1) the simulated structure, (2) conserved phosphorylation sites (e.g., S14) and surrounding areas, and (3) hydrophilic amino acid stretches. WWOX peptides were synthesized (>95% pure) by Genemed Synthesis (San Antonio, TX, USA). Synthetic WWOX peptides were: (1) WWOX7–21, NH-AGLDDTDSEDELPPG-COOH; (2) pS14-WWOX7–21, NH-AGLDDTDpSEDELPPG-COOH; (3) WWOX7–11, NH-AGLDD-COOH; (3) WWOX286-299, NH-DYWAMLAYNRSKLC-COOH; (4) pY287-WWOX286-299, NH-DpYWAMLAYNRSKLC-COOH. WWOX7–21 is in front of the *N*-terminal WW domain, and WWOX286-299 at the *C*-terminal SDR (short-chain alcohol dehydrogenase/reductase) domain. Scrambled WWOX7–11 peptides were: (1) NH-DLDGA-COOH; (2) NH-LDGDA-COOH; (3) NH-IGIDD-COOH; (4) NH-AGLEE-COOH. Zfra peptides were: (1) Zfra1-31, NH-MSSRRSSSCKYCEQDFRAHTQKNAATPFLAN-COOH; (2) Zfra4–10, NH-RRSSSCK-COOH [30,31]. These peptide stocks were made as 10 mM in degassed sterile Milli-Q water. Each tube was flushed with nitrogen gas and stored in −80 °C freezer. For tail vein injections in mice, peptides were freshly diluted at 1–4 mM in 100 μL degassed PBS.

### 4.3. Cancer Growth and Immune Cell Differentiation in Mice

We followed the US NIH guidelines and the approved protocols from the Intramural Animal Use and Care Committee (IACUC) of the National Cheng Kung University (NCKU) for animal experiments. 6–8 weeks old male BALB/c mice, NOD-SCID (NOD.CB17-*Prkdc^scid^*/NCrCrl) mice (Laboratory Animal Center, NCKU, Tainan, Taiwan), or nude (BALB/cAnN.Cg-*Foxn1^nu^*/CrlNarl) mice (National Laboratory Animal Center, Taibei, Taiwan) were used. Mice were inoculated with B16F10, 4T1, or other indicated cells in both left and right flanks and then received indicated WWOX or Zfra peptides (1 mM in 100 µL PBS) or PBS via tail vein injections one week later. Alternatively, tumor cells were allowed to grow up to 200 mm^3^ prior to peptide treatment. In addition, mice received intravenous injections of an indicated WWOX peptide to one side of the flanks and melanoma B16F10 to the other simultaneously. Tumor volumes were measured twice per week and calculated: *D* × (*d*)^2^/2, where *D* and *d* are the major and minor diameters, respectively. Tissue sections of spleen, lung and tumor lesions were prepared for immunohistochemistry using specific antibodies [30,31].

### 4.4. Antibodies, Immunohistochemistry, and Immunofluorescence Microscopy 

Commercial antibodies used were: polyclonal CD4, CD8α, CD8β, CD19, Foxp3, and monoclonal CD3, CD44, and WWOX from Santa Cruz Biotechnology (Dallas, Tx, USA). We made specific antibodies against WWOX286-299 and WWOX7-21, and phosphorylation at Y287, pS14 and pY33, respectively [12,16,17,33]. Also, we made antibodies against Hyal-2 using synthetic peptides at amino acids #211-226 and #227-241 [27,28,44]. A phosphopeptide at pY216-Hyal-2 (amino acids #211-226) was made for immunization and antibody generation in rabbits, followed by specific antibody purification and validation, according to our established procedures [16]. Immunohistochemistry and immunofluorescence microscopy were carried out using indicated tissue sections, according to our established procedures [30,31].

### 4.5. Time-Lapse Microscopy for 4T1 Stem Cell Sphere Explosion and Death

Where indicated, 4T1 breast cancer cell spheres were treated with one of the WWOX peptides for 30 min, followed by exposure to ceritinib. Time-lapse microscopy was carried out to determine the time course of 4T1 sphere explosion and death [28,33,38]. Where indicated, 4′,6-diamidino-2-phenylindole dihydrochloride (DAPI) and propidium iodide (PI) were added to measure the extent of nuclear membrane permeability changes and cell death, respectively.

### 4.6. Time-Lapse tri-Molecular Förster Resonance Energy Transfer (FRET) Microscopy

In addition to the standard two protein/protein binding FRET microscopy [28], we have recently developed thee-way protein/protein interaction FRET microscopy [28,33,38]. 4T1 cells were transiently transfected with ECFP-IκBα, EGFP-ERK, and DsRed-WWOX to determine tri-molecular binding interactions [28,33,38]. For time-lapse FRET microscopy, excitation of ectopic ECFP-IκBα protein allows energy transfer to EGFP-ERK for excitation, followed by subsequent energy transfer to DsRed-WWOX for excitation. 4T1 cells were transiently transfected with the aforementioned constructs, cultured for 24 to 48 h, and then treated with ceritinib (30 µM) for time-lapse FRET microscopy using an IX81 inverted fluorescence microscope (Olympus, Tokyo, Japan). Background fluorescence from an area without cells and spectral bleed-though were corrected. The FRET signals were directly visualized under the microscope and quantified as FRET concentration (FRETc) using the Olympus FRET analysis program. In negative control, cells were transfected with ECFP, EGFP and DsRed monomer. The profiles of time-related FRETc changes were analyzed by exponential regression analysis using Microsoft′s Excel software program.

### 4.7. Data Presentation and Statistical Analysis

In immunohistochemistry, five to six tissue sections were stained with a specific antibody and a representative data set is shown. Also, a representative data set from the kinetics of tumor cell growth in tumor-bearing mice is shown from two to six experiments, as specified.

### 4.8. Ethics Approval in Animal Use

All experiments involved in animal use have been approved by the Institutional Animal Care and Use Committee (IACUC) of the National Cheng Kung University College of Medicine (Approval numbers 105064, 106064, 107027 and 107208).

## 5. Conclusions

We have identified the *N*-terminal epitopes WWOX7-21 and WWOX7-11, which are involved in blocking breast cancer and melanoma growth in vivo. These peptides probably utilize the Hyal-2/WWOX/Smad4 signaling to exert cancer growth suppression and assist ceritinib-mediated enhancement of cancer cell death. However, when S14 is phosphorylated and Y33 is de-phosphorylated in WWOX, pS14-WWOX supports cancer growth. The specific enzyme for pY33 to pS14 transition is unknown. Nevertheless, the WWOX7-21 and WWOX7-11 peptides and pS14-WWOX antibody are of therapeutic vales in suppressing melanoma and breast cancer growth in vivo.

## Supplementary Materials

The following are available online at https://www.mdpi.com/2072-6694/11/11/1818/s1, Figure S1: WWOX is clustered in the cell membrane. Figure S2: WWOX is clustered in the cell membrane. Figure S3: Expression of pS14-WWOX in the cancer stem cell spheres. Figure S4: Stages in ceritinib-mediated 4T1 breast cancer stem cell sphere explosion and death. Figure S5: pS14-WWOX7-21 peptide strongly protects breast 4T1 stem cell spheres from explosion and death caused by ceritinib. Video S1: Ceritinib induces individual breast 4T1 cell apoptosis and stem cell sphere explosion and death; Video S2: WWOX286-299 antiserum did not block ceritinib-induced death of breast 4T1 cells. Video S3: WWOX7-21 antiserum blocked ceritinib-induced death of breast 4T1 cells. Video S4: Ceritinib induced death of breast 4T1 cells at room temperature. Video S5: WWOX7-21 peptide accelerated ceritinib-induced death of breast 4T1 cells at room temperature.
