# Supplementary material for: WWOX Possesses N-Terminal Cell Surface-Exposed Epitopes WWOX7-21 and WWOX7-11 for Signaling Cancer Growth Suppression and Prevention In Vivo
Source: Cancers (Basel). 2019 Nov 19;11(11):1818. doi: 10.3390/cancers11111818 (PMC6895976; doi:10.3390/cancers11111818)
Supplement: Supplementary file 1 [file cancers-11-01818-s001.zip › supple.pdf]

SUPPLEMENTARY MATERIALS

Figure S1. WWOX is clustered in the cell membrane. Live colon cancer HCT116 cells were stained with an indicated antibody at 4°C for 30 min, followed by staining with LavaCell for cell membrane. WWOX clusters on the cell surface. In negative controls, no primary antibody was added. WWOX(C) = Antibody against the *C* terminus of WWOX at amino acid #286 to 299. WWOX(N) = Antibody against the *N* terminus of WWOX at amino acid #20 to 42. pY33 is in the first WW domain, and pY287 in the SDR domain.

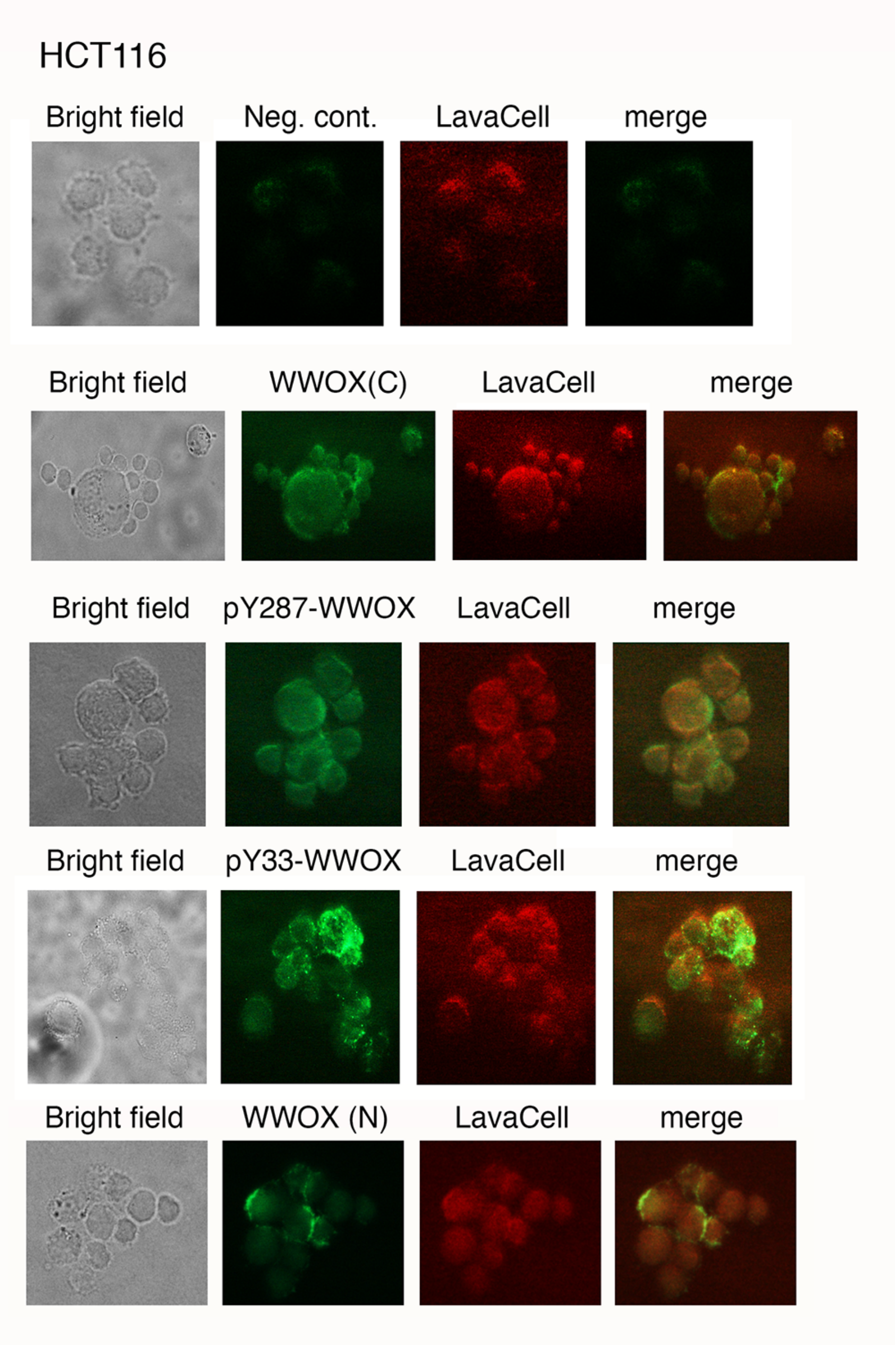

Figure S2. WWOX is clustered in the cell membrane. Live squamous cell carcinoma SCC4 cells were stained with an indicated antibody at 4ºC for 30 min, followed by staining with LavaCell for cell membrane. WWOX is clustered in cell surface and in the inclusion bodies. In negative controls, no primary antibody was added. WWOX(C) = Antibody against the *C* terminus of WWOX at amino acid #286 to 299. pY61 is in the second WW domain, and pT393 located in the *C*-terminal D3 tail [34].

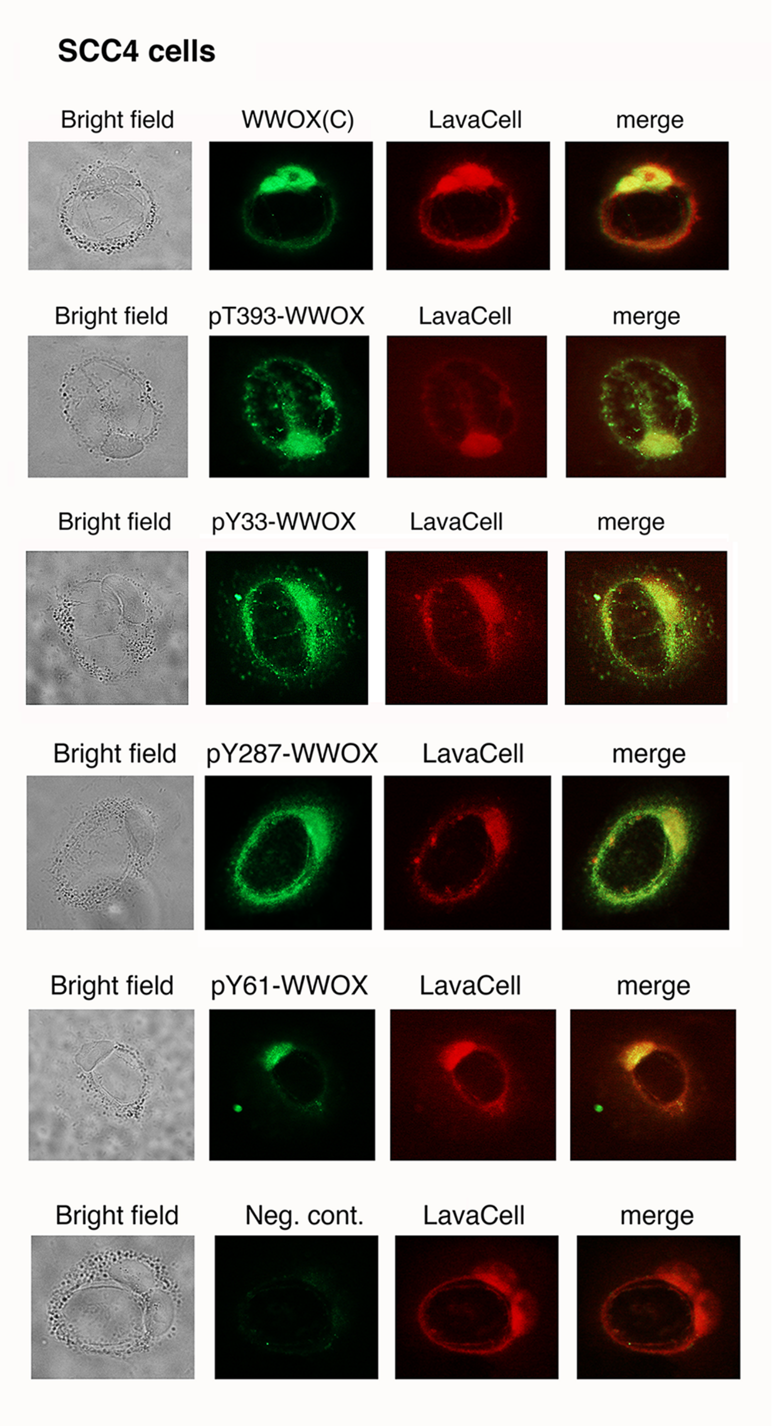

Figure S3. Expression of pS14-WWOX in the cancer stem cell spheres. Human NB69 neuroblastoma cells were cultured at 37°C for 24 to 48 hr. Formation of stem cell sphere occurred. Live cells in the inner mass of the spheres express stem cell markers such as SSEA-4 and TRA-1-60, as well as WWOX and its Ser14 phosphorylation. No pY287-WWOX expression is shown. Similar results were observed by examining mouse 4T1 breast cancer and HCT116 colon cancer cells (data not shown).

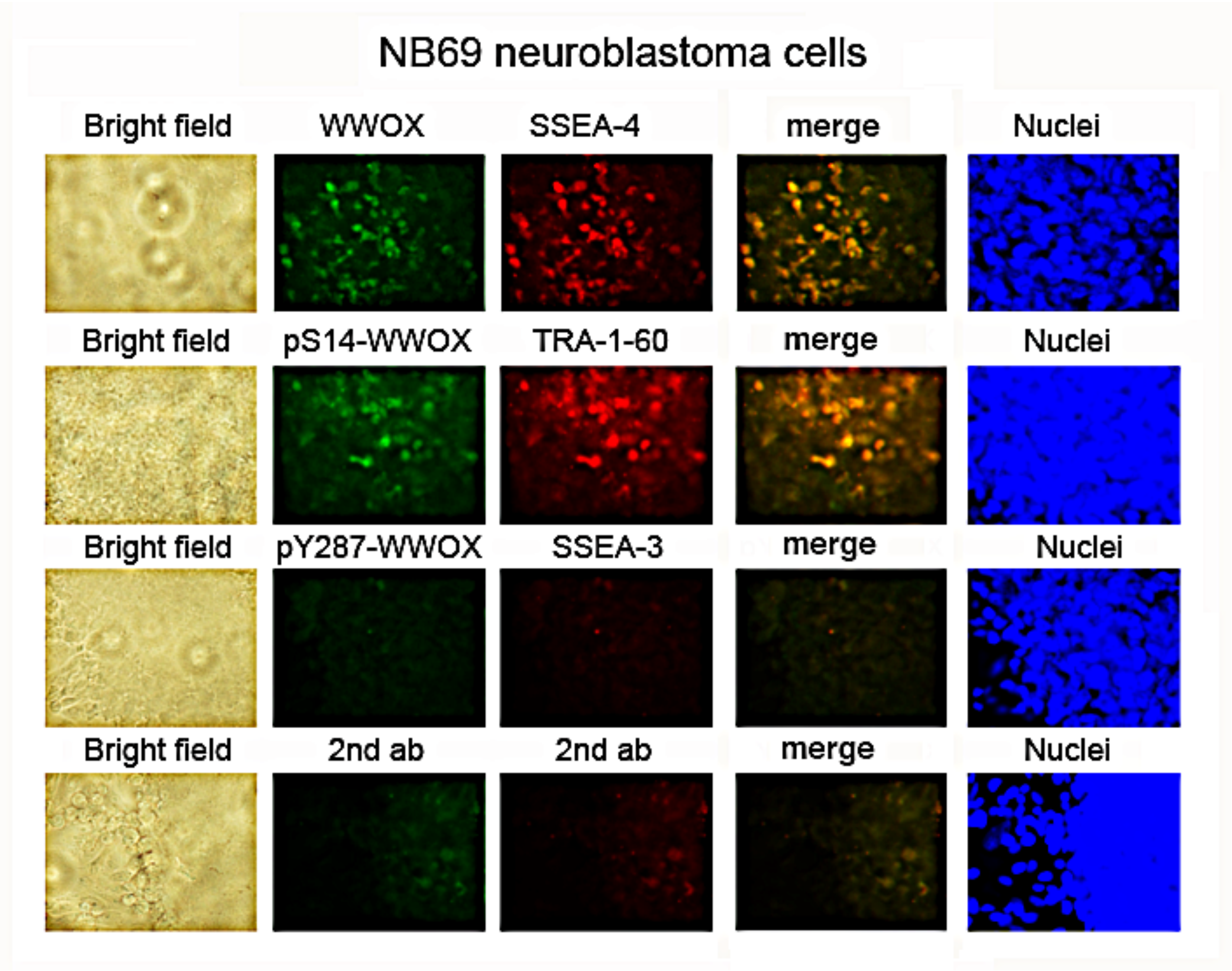

Figure S4. Stages in ceritinib-mediated 4T1 breast cancer stem cell sphere explosion and death. Breast 4T1 cells were cultured for 24 hr and then treated with ceritinib (30 μM) for time-lapse microscopy. The cell spheres underwent shrinkage (pre-explosion stage) and then explosion and death (explosion stage).

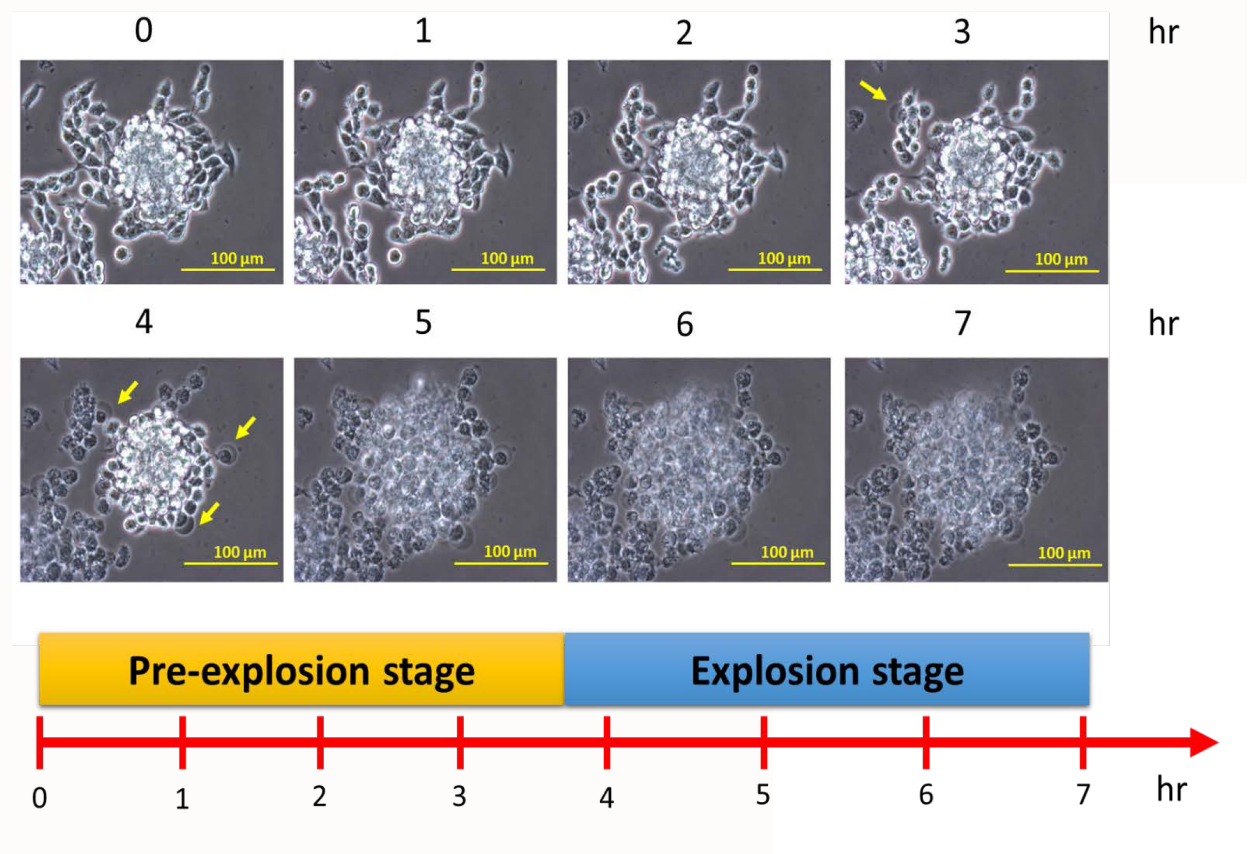

Figure S5. pS14-WWOX$_{7-21}$ peptide strongly protects breast 4T1 stem cell spheres from explosion and death caused by ceritinib. **(A)** At room temperature, ceritinib induced 4T1 stem cell sphere explosion and death (see the red stars for end points). pS14-WWOX$_{7-21}$ peptide strongly blocked ceritinib-mediated cell death, whereas WWOX$_{7-21}$ peptide accelerated the cell death. Cells surrounding the sphere underwent bubbling cell death [19,28,39]. **(B)** In an alternative experiment at 37°C, pS14-WWOX$_{7-21}$ peptide strongly blocked the sphere explosion and death caused by ceritinib (surviving longer than 48 hr).

A

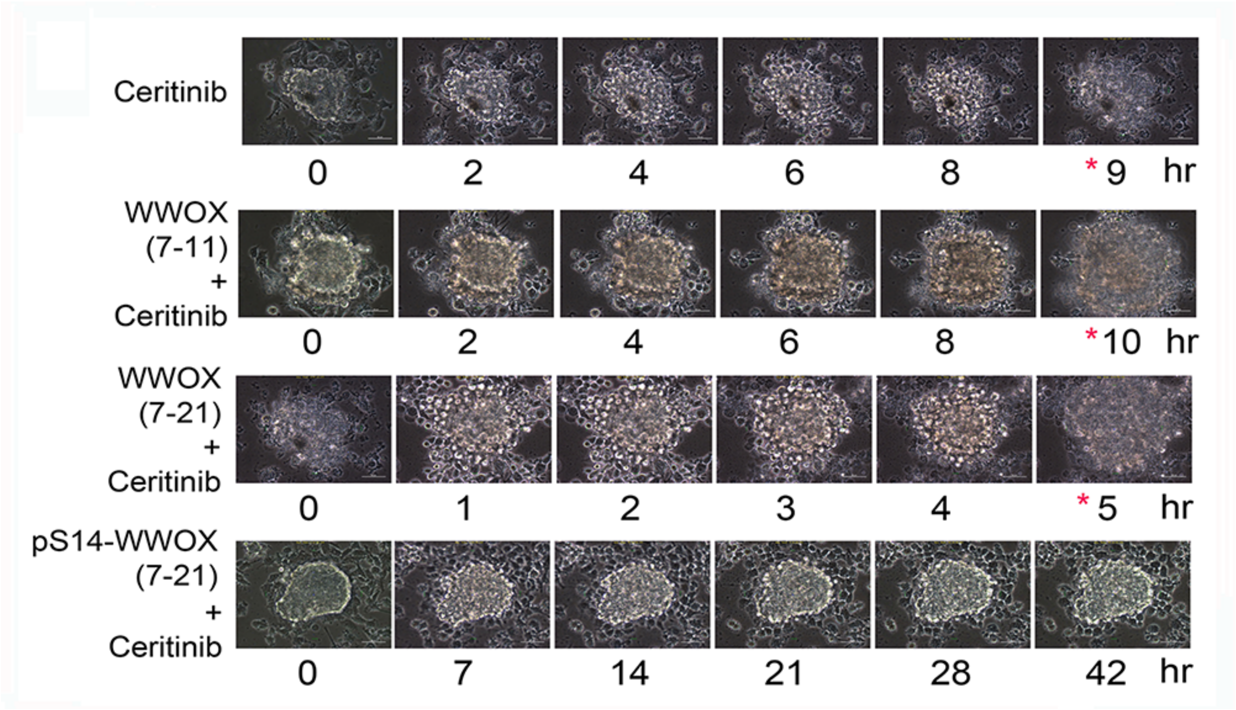

B

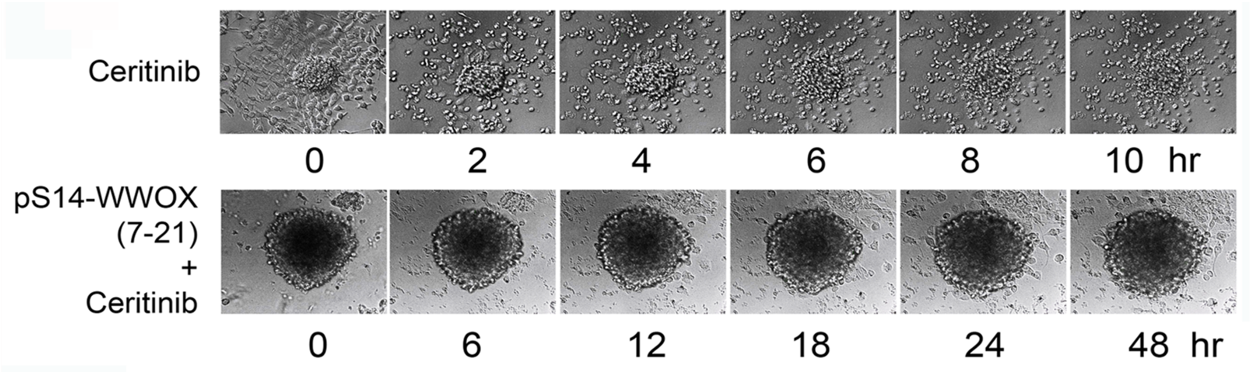

Supplemental Video Legends

Video S1. Ceritinib induces individual breast 4T1 cell apoptosis and stem cell sphere explosion and death. 4T1 cells were pretreated with 10 μl non-immune rabbit serum for 30 min, followed by treating with 30 μM ceritinib for imaging the cell death event by time-lapse microscopy (15 min per frame). Note that the cells picked up DAPI first (without death; blue) and then PI (red) in dead cells. The stem cell sphere underwent explosion and then death. (Data linked to Fig. 5a)

Video S2. $WWOX_{286-299}$ antiserum did not block ceritinib-induced death of breast 4T1 cells. Under similar conditions, 4T1 cells were pretreated with 10 μl WWOX antiserum against the $WWOX_{286-299}$ peptide for 30 min, followed by treating with 30 μM ceritinib for imaging of cell death by time-lapse microscopy (15 min per frame). (Data linked to Fig. 5a)

Video S3. $WWOX_{7-21}$ antiserum blocked ceritinib-induced death of breast 4T1 cells. Under similar conditions, 4T1 cells were pretreated with 10 μl WWOX antiserum against $WWOX_{7-21}$ peptide for 30 min, followed by treating with 30 μM ceritinib for imaging of cell death by time-lapse microscopy (15 min per frame). (Data linked to Fig. 5a)

Video S4. Ceritinib induced death of breast 4T1 cells at room temperature. At room temperature, 4T1 cells were treated with 30 μM ceritinib for imaging of cell death by time-lapse microscopy (10 min per frame). (Data linked to Fig. S5)

Video S5. $WWOX_{7-21}$ peptide accelerated ceritinib-induced death of breast 4T1 cells at room temperature. At room temperature, 4T1 cells were pretreated with $WWOX_{7-21}$ peptide and then treated with 30 μM ceritinib for imaging of cell death by time-lapse microscopy (10 min per frame). (Data linked to Fig. S5)
